# Antiviral Effects of Black Raspberry (*Rubus coreanus*) Seed and Its Gallic Acid against Influenza Virus Infection

**DOI:** 10.3390/v8060157

**Published:** 2016-06-06

**Authors:** Ji-Hye Lee, Mi Oh, Jong Hyeon Seok, Sella Kim, Dan Bi Lee, Garam Bae, Hae-In Bae, Seon Young Bae, Young-Min Hong, Sang-Oh Kwon, Dong-Hun Lee, Chang-Seon Song, Ji Young Mun, Mi Sook Chung, Kyung Hyun Kim

**Affiliations:** 1 Department of Biotechnology and Bioinformatics, Korea University, Sejong 30019, Korea; jihyelee@korea.ac.kr (J.-H.L.); tjrwhdgus@korea.ac.kr (J.H.S.); nawatogether@korea.ac.kr (S.K.); ann21010@korea.ac.kr (D.B.L.); 2 Department of Food and Nutrition, Duksung Women’s University, Seoul 01369, Korea; om5865@duksung.ac.kr (M.O.); rkfka3661@duksung.ac.kr (G.B.); haein1017@duksung.ac.kr (H.-I.B.); emily0605@nate.com (S.Y.B.); 3 R & D Center, Dong-il Shimadzu Corp., Seoul 08506, Korea; ymhong@shimadzu.co.kr; 4 S & D Co., Ltd., Osong, Cheongju, Chungbuk 28156, Korea; so-kwon0004@hanmail.net; 5 College of Veterinary Medicine, Konkuk University, Seoul 05029, Korea; chiclid@konkuk.ac.kr (D.-H.L.); songcs@konkuk.ac.kr (C.-S.S.); 6 Department of Biomedical Laboratory Science, College of Health Science, Eulji University, Gyeonggi-do 13135, Korea; jiyoung.mun@eulji.ac.kr

**Keywords:** influenza virus, *Rubus coreanus*, black raspberry seed, antiviral activity, gallic acid

## Abstract

Influenza is a serious public health concern worldwide, as it causes significant morbidity and mortality. The emergence of drug-resistant viral strains requires new approaches for the treatment of influenza. In this study, *Rubus coreanus* seed (RCS) that is left over from the production of wine or juice was found to show antiviral activities against influenza type A and B viruses. Using the time-of-addition plaque assay, viral replication was almost completely abolished by simultaneous treatment with the RCS fraction of less than a 1-kDa molecular weight (RCSF1). One of the polyphenols derived from RCSF1, gallic acid (GA), identified by liquid chromatography-tandem mass spectrometry, showed inhibitory effects against both influenza type A and B viruses, albeit at relatively high concentrations. RCSF1 was bound to hemagglutinin protein, inhibited hemagglutination significantly and disrupted viral particles, whereas GA was found to only disrupt the viral particles by using transmission electron microscopy. In BALB/c mice infected with influenza virus, oral administration of RCSF1 significantly improved the survival rate and reduced the viral titers in the lungs. Our results demonstrate that RCSF1 and GA show potent and broad antiviral activity against influenza A and B type viruses and are promising sources of agents that target virus particles.

## 1. Introduction

Influenza virus is a single-stranded RNA virus, which belongs to the *Orthomyxoviridae* family, and causes acute respiratory infection responsible for seasonal epidemics and occasional pandemics. There are three types of influenza viruses: A, B and C. Type A virus consists of eight single-stranded RNAs encoding 13 proteins [1] and is divided into subtypes according to the antigenic properties of surface glycoproteins, hemagglutinin (HA) and neuraminidase (NA). Three classes of antiviral drugs, M2 inhibitors, NA inhibitors and polymerase inhibitors, have proven effective in preventing influenza viral infection [2]. Despite the success of these drugs, concerns remain regarding drug efficacy, resistance and cost [3].

Plant extracts and constituents have been reported to have antiviral activities against influenza viruses: Aronia, green tea, green tea by-products, dandelion and red algal lectin [4,5,6,7,8]. Many polyphenols have been also studied for their antiviral activities, including ellagic acid, theaflavin derivatives and catechin [4,9,10]. However, our understanding of the potential of plant extracts is still limited, despite nature’s abundant source of chemical diversity that has evolved to bind biological targets and elicit biological effects. In addition, obtaining sufficient quantities of the extracts is a major hurdle.

*Rubus coreanus* (RC), which belongs to the *Rosaceae* family, is a species of black raspberry native to Korea, Japan and China [11]. RC is rich in polyphenols, having the highest antioxidant capacity among fruits and vegetables, and its juice was reported to possess antibacterial activity [12,13,14]. Importantly, RC seeds, which are left over from wine or juice production, also contain large quantities of polyphenols [13]. However, the antiviral activities of RC seed against influenza viruses have not been explored.

Herein, we investigated the antiviral effects of the low molecular weight fraction (RCSF1) of RC seed extract (RCS) and its polyphenol, gallic acid (GA), against influenza virus A and B strains in *in vitro* assays. RCSF1 significantly reduced viral attachment and disrupted viral particle, whereas GA was found to disrupt the viral particles only. RCSF1 was further shown to have high *in vivo* efficacy.

## 2. Materials and Methods

### 2.1. Viruses

The influenza strains, A/Brisbane/59/2007(H1N1) (BR59), pandemic A/Korea/01/2009(H1N1) (KR01), A/Brisbane/10/2007(H3N2) (BR10), B/Florida/04/2006 (FL04) and A/Puerto Rico/8/1934 (H1N1) (PR8), were obtained from the Korea Centers for Disease Control and Prevention. The viral stocks were prepared according to the WHO manual in the virus growth medium [15].

### 2.2. Preparation of RCSF1

The fine powder of freeze-dried RC seeds was extracted in 70% ethanol using ultrasonic sound (50 kHz, Hwashin Instrument, Bucheon, Korea) for 20 min at 20 °C and was centrifuged (9500 g, 60 min, 4 °C). The supernatant was loaded onto a 1-kDa molecular weight cut-off stirred ultrafiltration cell (Millipore Corporation, Billerica, MA, USA) and lyophilized (RCSF1).

### 2.3. Cytotoxicity

Cytotoxicity was measured by the 3-[4,5-dimethylthiazol-2-yl]-2,5-diphenyltetrazolium bromide (MTT) assay [16]. MDCK cells (1.5 × 10^4^ cells/well) from American Type Culture Collection (Manassas, VA, USA) were treated with RCSF1 for 24 h at 37 °C in a 5% CO_2_. The percentage of cell viability was calculated as follows: % cell viability = (Abs_treatment_ / Abs_control_) × 100.

### 2.4. Time-of-Addition Plaque Reduction Assay

For pre-virus treatment, virus (5 log_10_ plaque forming units (PFU)/mL) was pre-incubated with each concentration of RCSF1 for 1 h, and the serially diluted virus-RCSF1 mixture was added onto MDCK cells at 37 °C for 1 h in a 5% CO_2_ [17]. For co-treatment, cells were infected with virus (2–3 log_10_PFU/mL) and RCSF1 or polyphenols simultaneously for 1 h at 37 °C. For post-treatment, virus (2–3 log_10_PFU/mL) was absorbed to cells at 37 °C for 1 h. Cells were then treated with RCSF1 for 1 h. After each treatment, the cells were overlaid with Dulbecco’s Modification of Eagle’s Medium (DMEM; Gibco BRL, Karlsruhe, Germany) containing 1 μg/mL tosylsulfonyl phenylalanyl chloromethyl ketone (TPCK)-trypsin and 1.5% agarose for 72 h. Plaques were counted after 0.5% crystal violet staining. Oseltamivir (Hoffmann-La Roche, Basel, Switzerland) was used as the positive control.

### 2.5. Confocal Microscopy

MDCK cells were infected with BR59 virus at a multiplicity of infection (MOI) of 2. MOI was defined as the ratio of PFU of virus used for infection to the number of MDCK cells. After the treatment with RCSF1, cells were fixed and permeabilized with 0.3% Triton X, and nucleoprotein (NP) was detected by influenza A NP-specific monoclonal antibody (Santa Cruz Biotechnology, Santa Cruz, CA, USA) and Alexa Fluor 488-conjugated goat anti-mouse IgG (Sigma-Aldrich, St. Louis, MO, USA). 4′,6-Diamidino-2-phenylindole (DAPI) staining for nucleic acid was used as the control. Images were taken using a confocal laser scanning microscope (LSM 700, Carl Zeiss, Thornwood, NY, USA).

### 2.6. Hemagglutination Inhibition Assay

Two-fold serial diluted RCSF1 (final conc. 0.5–50 μg/mL) was prepared in 96-well plates as previously described [15]. Serially-diluted RCSF1 was mixed and incubated with the same volume of virus (25 μL/well containing 4 hemagglutinating units) at room temperature for 15 min. Additionally, 50 μL of 0.5% chicken red blood cells were added and incubated for 1 h before hemagglutination inhibition was determined.

### 2.7. Differential Scanning Fluorometry and Fusion Inhibition Assay

Purified HA (0.5 mg/mL) prepared as described previously [18] was incubated with RCSF1 (10–200 µg/mL) for 30 min and mixed with 1 μL SYPRO Orange. The temperature increment at the rate of 0.5 °C every 30 s for 50 min and the melting temperature (T_m_) were calculated from the maxima of the first derivative of relative fluorescence units/temperature using Mx3005P DSF and MxPro QPCR Software (Stratagene, CA, USA).

The syncytium formation assay was performed as described previously [19]. Monolayers of Vero cells were infected with PR8 virus at an MOI 3 in the presence or absence of RCSF1. At 6 h post-infection, cells were washed twice with PBS containing Ca^2+^/Mg^2+^ and exposed for 5 min to pH 5.0 at 37 °C, which were neutralized and incubated for 2 h. Cells were fixed and stained with a Hema 3 stat pack staining kit (Fisher Scientific, Waltham, MA, USA). Syncytium formation was observed by inverted microscope.

### 2.8. Attachment and Penetration Assays

Chilled MDCK cells were infected with virus (2–3 log_10_PFU) and simultaneously treated with RCSF1 (0.5–50 μg/mL) for 1 h at 4 °C, which allowed for virus attachment, but prevented penetration [20]. After infection, the supernatant was removed, and cells were washed twice with ice-cold PBS. For the penetration assay [20], the chilled MDCK cells were infected with virus at 4 °C and treated with RCSF1 (0.5–50 μg/mL) at 37 °C for 1 h. After the supernatant was removed, un-penetrated virus was inactivated by acidic PBS (pH 3) and neutralized with alkaline PBS (pH 11). After washing with ice-cold PBS, the cells were overlaid with DMEM containing 1 μg/mL TPCK-trypsin and 1.5% agarose for 72 h at 37 °C, 5% CO_2_ incubator. Plaques were counted after 0.5% crystal violet staining. Virus inhibition was evaluated by the plaque reduction assay.

### 2.9. NA Treatment on RCSF1

The effect of NA treatment on RCSF1 was analyzed through hemagglutination inhibition (HI) and cytopathic effect (CPE) [20]. RCSF1 (1 μg) was incubated with NA (1 unit; Sigma-Aldrich) for 20 h at 37 °C, which was then inactivated at 56 °C for 60 min and subjected to the HI assay. MDCK cells were infected with virus at an MOI of 0.001 for 1 h at 37 °C. After removing the medium, the cells were treated with RCSF1 or NA-treated RCSF1, which was serially diluted in VGM containing 1 μg/mL TPCK-trypsin. After incubation for 72 h at 37 °C and 5% CO_2_, the cell viability was measured using the MTT assay.

### 2.10. Transmission Electron Microscopy

BR59 virus (6 log_10_PFU/mL) was incubated with RCSF1 or GA (Sigma-Aldrich) for 1 h at room temperature, which was applied to a freshly-charged carbon-coated grid and stained with uranyl acetate solution. The stained viruses were observed with a Hitachi H7600 transmission electron microscope (Hitachi, Tokyo, Japan) at 80 kV at a magnification of 20,000.

### 2.11. Identification of Polyphenols by LC/MS/MS

Polyphenols of RCSF1 were quantitatively analyzed using a LCMS-8040™ liquid chromatography mass spectrometer (Shimadzu, Kyoto, Japan) with an electrospray ionization probe and a Nexera UHPLC system equipped with a phenyl-hexyl column (2.1 × 100 mm, 3.5 μm; Agilent, Santa Clara, CA, USA). Detailed multiple reaction monitoring conditions of target analytes are indicated in Table S1. Standard polyphenols purchased from Sigma-Aldrich were identified as previously reported [11].

### 2.12. In Vivo Mouse Experiments

All animal experiments were performed in accordance with the recommendations in the Guide for the Care and Use of Laboratory Animals from the Animal, Plant and Fisheries Quarantine and Inspection Agency, Republic of Korea. The study protocol was approved by the Institutional Animal Care and Use Committee of Duksung Women’s University (Approval Number DSU13-015-008). All efforts were made to reduce suffering of the animals. For *in vivo* toxicity of RCSF1, six-week-old BALB/c mice (five per group) from Koatech (Kyunggi-do, Korea) were orally treated with RCSF1 at doses of 0, 3.5, 15 and 50 mg/kg/day for 8 days. Body weight changes of mice were measured daily for 14 days. For the antiviral activity of RCSF1, six groups (eight per group) were assigned: naive, negative control (PBS), positive control (oseltamivir phosphate, 0.75 mg/kg/day; Hoffmann-La Roche) and RCSF1 (1, 3.5 and 15 mg/kg/day). Mice were anesthetized and intranasally infected with three-times the 50% mouse lethal dose (3 MLD_50_) of mouse-adapted PR8 virus. The infected mice were administered orally once per day for 5 days with the indicated concentrations of RCSF1. The body weight of mice was measured for daily weight loss, and mice were sacrificed upon a body weight loss of 20% at the utmost. Three mice from each group were sacrificed at 3 days post-infection (dpi), and endpoint lung virus titers were determined as TCID_50_/mL from triplicate. Briefly, homogenates of the lung tissue were centrifuged at 400× *g* and 4 °C for 5 min. The supernatant was collected to be the lung suspension. MDCK cell monolayers were infected for 1 h with 50 μL of serial 1:10 dilutions of the lung suspension in a 96-well plate. Following inoculation, the supernatant was replaced by DMEM containing 2 mg/mL trypsin and incubated for 72 h at 37 °C in a 5% CO_2_ incubator. CPE was observed under light microscopy. The TCID_50_ value was obtained through calculating by the Reed–Muench method [21].

### 2.13. Statistical Analysis

All measurements were carried out in triplicate. Experimental results were expressed as the mean ± SD. Data were analyzed using analysis of variance (ANOVA) with SPSS software (Version 13.0, SPSS Inc., Chicago, IL, USA), and the means were separated by Duncan’s multiple range test. Values of *p* < 0.05 were considered statistically significant.

## 3. Results

### 3.1. RCSF1 Inhibits Early Stages of Influenza Viral Infection

**Figure 1:**
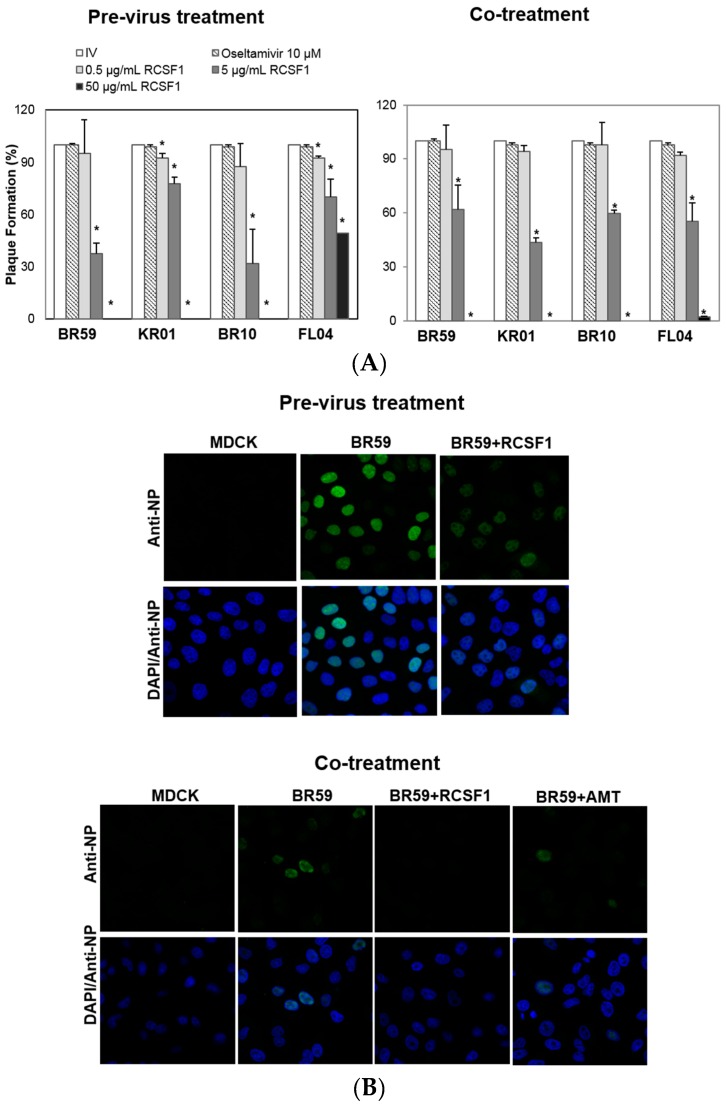
Antiviral activities of *Rubus coreanus* seed fraction 1 (RCSF1) against BR59, KR01, BR10 and FL04. In time-of-addition assays: (**A**) pre-virus treatment: each virus was mixed with RCSF1 for 1 h prior to viral infection; co-treatment: cells were infected with virus and RCSF1 simultaneously for 1 h at 37 °C. After infection, the cells were washed and overlaid with agarose at 37 °C for 72 h. Non-treated influenza virus (IV) and oseltamivir (10 μM) were used as negative and positive controls, respectively. All measurements were performed in triplicate. * *p* < 0.05. For confocal microscopy, BR59 was infected into MDCK cells at an MOI 2. Treatment of RCSF1 (50 µg/mL) was analyzed in (**B**) the pre-virus and co-treatment. Antiviral effect was analyzed by detection of viral NP (green) and DAPI (blue) using a confocal laser scanning microscope. Amantadine (AMT) was used as the positive control.

All assays were conducted at concentrations of RCS and RCSF1 without cytotoxicity (Figure S1). RCS at 50 μg/mL exhibited almost complete inhibitions of plaque formation of BR59, KR01 and BR10 by 93%–98% (Figure S2). It was fractionated to produce a fraction with a molecular weight less than 1 kDa, RCSF1. The antiviral effect of RCSF1 was examined at different time points during infection. The pre-virus treatment of RCSF1 at 50 μg/mL exhibited complete inhibitions against type A viruses, BR59, KR01 and BR10 (Figure 1A). The co-treatment of RCSF1 at 50 μg/mL resulted in almost complete abolishment of plaque formation against both the A influenza viruses and type B virus, FL04 (Figure 1A). In the post-treatment assay, however, the inhibitory effect of RCSF1 was weak, compared to that in the pre- or co-treatment (Figure S3). Oseltamivir, a neuraminidase inhibitor, that prevents the virus from being released from infected cells, also showed an antiviral activity at the post-treatment. Confocal microscopic studies also showed that in the pre-virus and the co-treatment, RCSF1 reduced the viral NP protein signal intensities in cells (Figure 1B).

### 3.2. RCSF1 Inhibits Hemagglutination and Fusion and Disrupts Virus Particles

**Figure 2:**
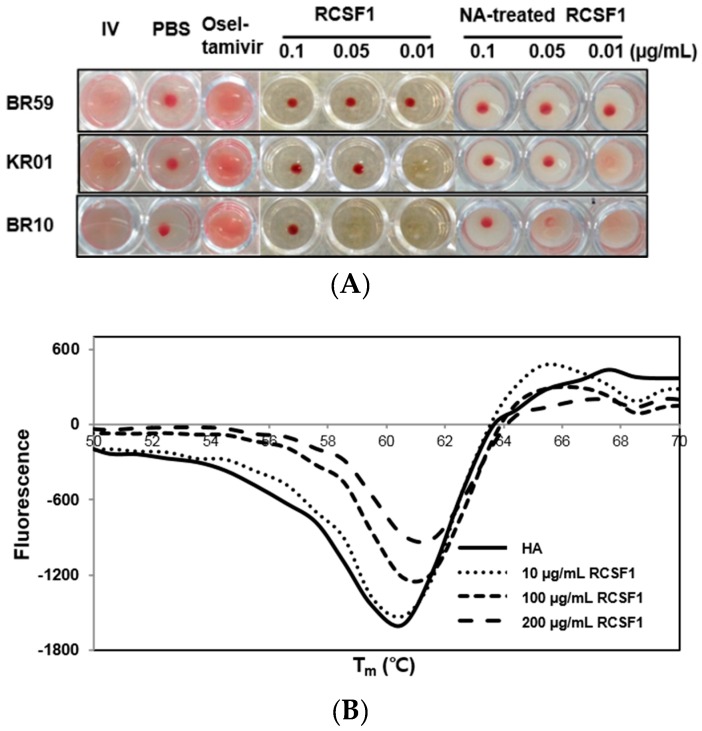

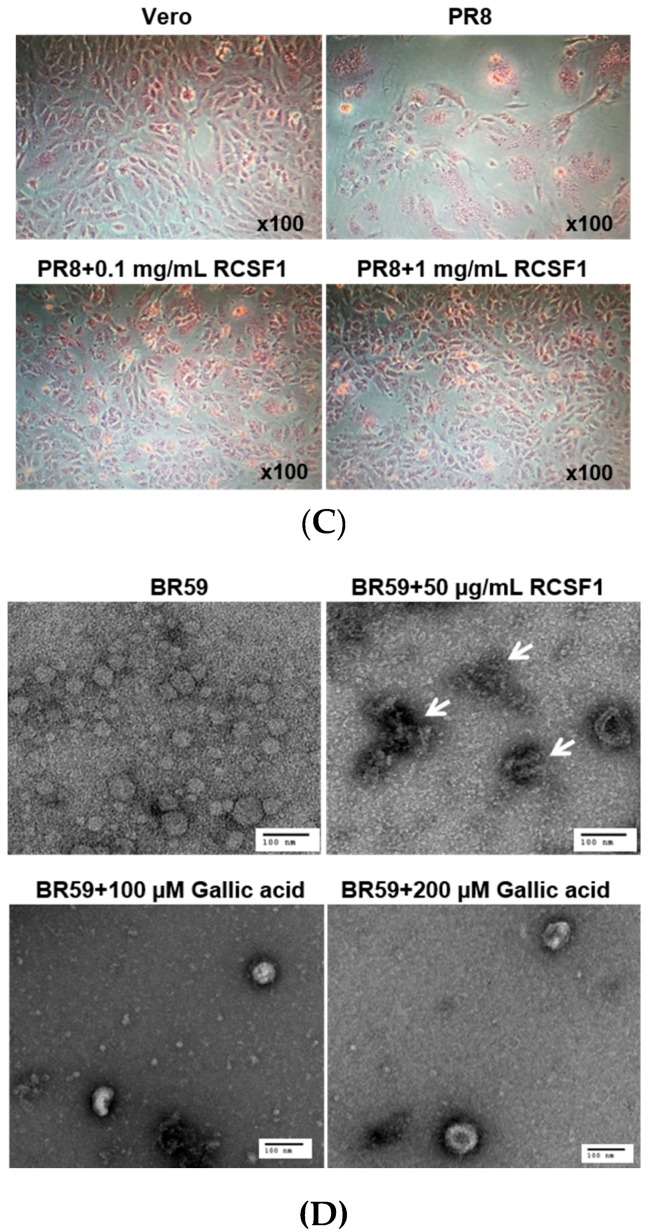
Inhibitory effects of RCSF1 or GA at the early stage of viral infection. (**A**) Hemagglutination inhibition assay. Diluted RCSF1 or NA-treated RCSF1 were incubated with virus and 0.5% chicken red blood cells for 1 h; (**B**) Differential scanning fluorometry profile of influenza virus HA in the absence and presence of RCSF1; (**C**) Effect of RCSF1 on syncytium formation in PR8-infected Vero cells; (**D**) Effects of RCSF1 or GA on virus particles based on TEM images. White arrows indicate disrupted virus particles. Scale bars, 100 nm.

HA plays crucial roles at the early stage of virus infection. The HI assay showed that RCSF1 inhibited hemagglutination significantly, reaching the minimum inhibitory concentration of 0.01–0.1 μg/mL (Figure 2A). Using the DSF assay, RCSF1 (10–200 μg/mL) was found to increase the T_m_ of HA by 1 °C (Figure 2B). These results suggest that RCSF1 inhibits receptor-binding of HA and binds to HA directly. Next, the membrane fusion inhibition assay showed a significant inhibition of syncytium formation by RCSF1 at concentrations of 0.1–1 mg/mL (Figure 2C). RCSF1 was found to reduce virus attachment or penetration in a dose-dependent manner (Figure S4).

The primary receptor for influenza virus is a terminal sialic acid recognized by HA. However, sialic acids seem to have appeared late in evolution and are not generally found in plants, prokaryotes and invertebrates [22]. When the effects of NA-treated and non-treated RCSF1 on CPE and HI were compared, no significant difference was observed in cell viability (Figure S5) and HI assays (Figure 2A).

We then conducted direct observation of influenza virus particles by TEM. Disruption of virus particles was observed at the RCSF1 treatment, compared to the control (Figure 2D). Taken together, RCSF1 was found to bind to HA and to disrupt viral particles, which may inhibit receptor-binding of HA and syncytium formation, suggesting an inhibition at the early stage of viral infection.

### 3.3. Polyphenols of RCSF1, GA, Have Anti-Influenza Activities against Various Strains

Polyphenols in RCSF1 were quantitatively analyzed using LC/MS/MS. RCSF1 contained in decreasing order catechin, ellagic acid, cyanidin-3-rutinoside, 3,4-dihydroxybenzoic acid and GA, in the range of 1.3–34.6 mg/g, and other minor compounds (Table 1). Single polyphenols showed less significant antiviral activities against influenza A and B viruses than GA at concentrations from 1–100 μM (Figure S6).

**Figure 3:**
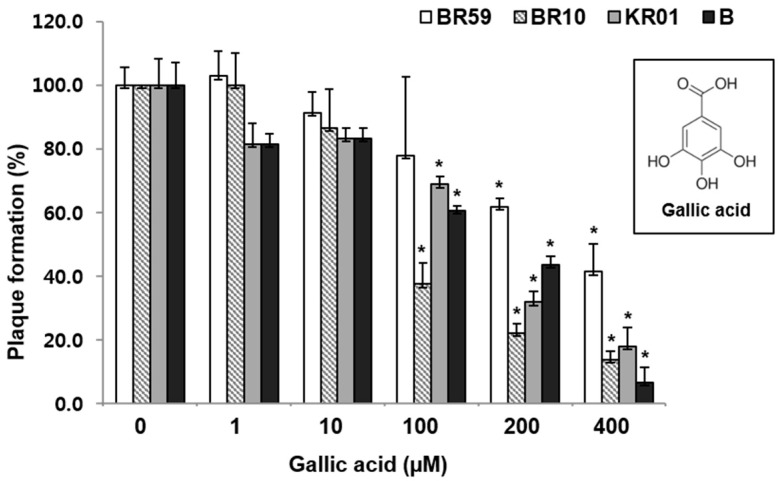
Antiviral effects of GA against influenza A and B viruses. MDCK cells were infected with virus and simultaneously treated with GA for 1 h at 37 °C. After infection, the cells were washed and overlaid with agarose at 37 °C for 72 h. Non-treated influenza virus was used as a negative control. All measurements were performed in triplicate. * *p* < 0.05.

GA was found to reduce the plaque formation by 59%–93% in a concentration-dependent manner in the range of 1–400 µM (Figure 3). As shown in the RCSF1 treatment, TEM images showed that GA was found to enlarge and disrupt viral particles (Figure 2D). However, no significant difference was observed in HI and DSF assays after the treatment of GA (Figure S7).

### 3.4. Oral Administration of RCSF1 Shows Anti-Influenza Activity in Mice

**Figure 4:**
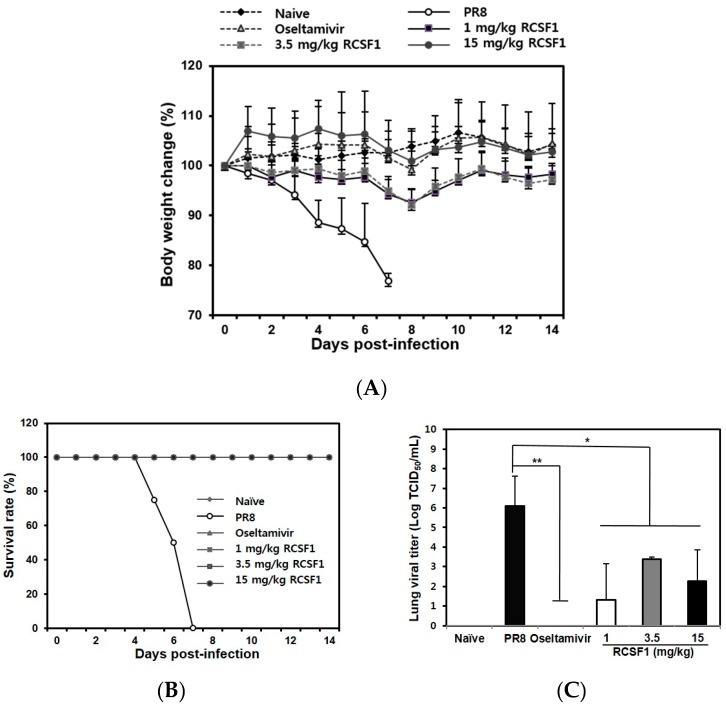
*In vivo* efficacy of RCSF1. Six groups (eight per group) were assigned: naive, negative control (PBS), positive control (oseltamivir phosphate, 0.75 mg/kg/day) and RCSF1 (1, 3.5, and 15 mg/kg/day). (**A**) Body weight changes; (**B**) survival rate until 14 dpi; and (**C**) viral lung titers by TCID_50_ at 3 dpi in BALB/c mice infected with influenza virus. * *p* < 0.05; ** *p* < 0.01.

RCSF1 (50 mg/kg/day) was not toxic in BALB/c mice (Figure S8). The RCSF1-treated mice (1, 3.5 and 15 mg/kg) did not show significant body weight loss, whereas mice in the negative control group started to show a clear body weight loss after 3 dpi (Figure 4A). All infected mice succumbed to death at 7 dpi. In contrast, RCSF1 exhibited full protection with a 100% survival rate, showing comparable activity with oseltamivir phosphate (Figure 4B). Furthermore, virus titers in the lungs were significantly reduced in the RCSF1-treated mice, significantly reducing lung viral titers at least two to four orders of magnitude at the concentration of above 1 mg/kg RCSF1 (Figure 4C). The results suggest that RCSF1 could decrease the influenza virus distribution in the lungs of mice.

## 4. Discussion

Current therapeutic agents for the prevention and treatment of influenza include NA inhibitors, M2 channel blockers and polymerase inhibitors [2]. However, use of these drugs suffers from limitations in their efficacy due to drug resistance [3,23]. Importantly, seasonal vaccines have shown limited efficacy in the elderly and in infection with zoonotic strains that cause a serious threat to human health [24]. There is a pressing need to develop new antiviral approaches for the treatment of this disease, and broad-spectrum antiviral drugs will provide alternative anti-influenza therapies. In this study, RCSF1 revealed strong inhibitory activities against type A and B influenza viruses, including KR01, a 2009 pandemic strain, when the virus was pre- or co-incubated with RCSF1. The major antiviral drugs target influenza virus enzymes [2]. HA is responsible for the entry and fusion of virions into host cells. Novel inhibitors blocking HA-mediated receptor binding or membrane fusion were previously reported [25,26]. A quinone compound binds to a hydrophobic pocket of HA, and thiazolidine inhibitors bind to virions to inhibit lipid mixing and to produce reactive oxygen species that disrupt the biophysical properties of viral membrane. Our results demonstrated that RCSF1 inhibited hemagglutination and directly bound to HA. It is nevertheless unlikely that RCSF1 from black raspberry seed contains sialic acid, since the presence of sialic acids in plants has not been identified [22]. In fact, NA-treated RCSF1 showed an essentially identical antiviral activity to that of the untreated one. In this context, RCSF1 may contain a receptor-like component, which is not recognized by NA, or different components, which mediate receptor binding activity. In addition, RCSF1 disrupted viral particles. GA, one of the RCSF1 components, which did not inhibit hemagglutination and binds to HA, was found to disrupt the virus particles in TEM results. It further showed plaque reduction activity, albeit at relatively high concentrations. Taken together, RCSF1 disrupted viral particles and showed inhibition against the adsorption and penetration of virus, suggesting either that RCSF1 damages the virion and subsequently inhibits virus adsorption and penetration or that it can show multiple inhibitory effects on virions, as well as virus adsorption and penetration. Further studies are in progress to evaluate the antiviral effects of the combination of GA and other polyphenols or GA derivatives. Plant extracts have been reported to inhibit influenza virus: this inhibitory activity is associated with the prevention of virus entry into host cells [8], the inhibition of viral polymerase activity [7] and the inhibition of transcription and release of virus [6]. Fractionation of RCSF1 indicates the presence of multiple polyphenols. Previous reports have shown inhibitory activities against influenza, including ellagic acid, epigallocatechin gallate and punicalagin [4,10,27]. In contrast, catechin, caffeic acid and ellagic acid have little or no anti-influenza activities [10,27]. The co-treatment with quercetin exerted a weak inhibition, but the pre-treatment of virus led to a significant inhibition on H1N1 [28]. In our plaque assay, the co-treatment with quercetin, one of minor compounds in RCSF1, at 1–100 µM showed little inhibitory effect against influenza A and B viruses. It was shown that the *Hamamelis virginiana* bark extract showed higher antiviral activity than any of its single polyphenols, including GA [29]. GA, a trihydroxybenzoic acid, among the polyphenols in this study, showed concentration-dependent inhibitory activities against both influenza A and B viruses. Two benzoic acid derivatives were reported to show antiviral potency against influenza A virus [30,31]. The GA content of RCSF1 was only 0.13%, compared to the major polyphenol components, catechin (3.5%) and ellagic acid (1.1%). Whereas catechin and ellagic acid did not show a significant inhibitory activity against influenza viruses, the minor component, gallic acid, showed a concentration-dependent inhibition at relatively high concentrations. In this context, the polyphenols identified in this study may not include all the components of RCSF1, suggesting that other polyphenols or other components in RCSF1 can be the major contributor to the antiviral activity. Further studies are required.

In the animal study, RCSF1 was proven to increase the survival rate of infected mice. Oral administration of RCSF1 significantly reduced the levels of lung viral titers at 3 dpi. Importantly, a very low dose (1 mg/kg body weight) of RCSF1 not only reduced virus loads in the lungs, but also protected the mice from challenges with PR8 virus. Taken together, RCSF1 and GA showed inhibitory effects with a different mode-of-action at the early stage of both type A and B viral infections and they can be an attractive candidate for development as an antiviral agent against influenza viruses.

## 5. Conclusions

RCSF1 and GA showed potent and broad antiviral activity against influenza A and B type viruses, and treatment with an oral administration of RCSF1 in mice was able to provide protection against challenge with PR8 at the concentration of above 1 mg/kg.

## Figures and Tables

**Table 1:** Contents of polyphenols in RCSF1 by LC/MS/MS (mg/g).

Class	Polyphenols	Contents
Flavonoids	Cyanidin-3-glucoside	0.15 ± 0.00
	Cyanidin-3-rutinoside	8.50 ± 0.20
	Quercetin	0.27 ± 0.01
	Myricetin	ND ^1^
	Rutin	0.53 ± 0.00
	Catechin	34.55 ± 0.82
	Epigallocatechin gallate	ND
	Ellagic acid	11.43 ± 0.99
Phenolic acid	Gallic acid	1.30 ± 0.04
	3,4-Dihydroxybenzoic acid	1.99 ± 0.05
	Caffeic acid	0.22 ± 0.03
	*trans*-Ferulic acid	0.13 ± 0.01
	*p*-Coumaric acid	0.44 ± 0.00
	Chlorogenic acid	0.02 ± 0.00
Stilbenoids	*trans*-Resveratrol	0.02 ± 0.00

^1^ ND: not detected.

## Supplementary Materials

The following are available online at www.mdpi.com/1999-4915/8/6/157/s1, Table S1: Multiple reaction monitoring conditions of polyphenols from RCSF1, Figure S1: Cytotoxicity of RCS and RCSF1, Figure S2: Antiviral activities of RCS against BR59, KR01, BR10, and FL04, Figure S3: Antiviral activities of RCSF1 against BR59, KR01, BR10, and FL04 in the post-treatment, Figure S4: Inhibitory effects of RCSF1 on attachment or penetration into host cells, Figure S5: Effect of NA treatment on RCSF1, Figure S6: Antiviral activities polyphenols against influenza type A and B in the co-treatment, Figure S7: Effects of gallic acid on hemagglutination and binding to hemagglutinin, Figure S8: *In vivo* toxicity of RCSF1.
