# Supplementary material for: Antiviral Effects of Black Raspberry (Rubus coreanus) Seed and Its Gallic Acid against Influenza Virus Infection
Source: Viruses. 2016 Jun 6;8(6):157. doi: 10.3390/v8060157 (PMC4926177; doi:10.3390/v8060157)
Supplement: Supplementary file 1 [file viruses-08-00157-s001.pdf]

Supplementary Materials: Antiviral Effects of Black Raspberry (*Rubus coreanus*) Seed and its Gallic Acid against Influenza Virus Infection

Ji-Hye Lee, Mi Oh, Jong Hyeon Seok, Sella Kim, Dan Bi Lee, Garam Bae, Hae-In Bae, Seon Young Bae, Young-Min Hong, Sang-Oh Kwon, Dong-Hun Lee, Chang-Seon Song, Ji Young Mun, Mi Sook Chung and Kyung Hyun Kim

Table S1. Multiple reaction monitoring conditions of polyphenols from RCSF1.

Analyte	Retention Time (min)	Exact Mass	Ionization	Precursor	Product 1	Collision Energy (V)	Product 2	Collision Energy (V)
Caffeic acid	4.29	180.04	$[M-H]^-$	179	135	16	134	25
Catechin	3.96	290.08	$[M+H]^+$	291	139	−15	123	−16
Chlorogenic acid	4.07	354.10	$[M-H]^-$	353	191	16	85	45
p-Coumaric acid	5.20	164.05	$[M-H]^-$	163	119	15	93	31
Cyanidin-3-glucoside	3.74	448.10	$[M+H]^+$	449	287	−22	137	−55
Cyanidin-3-rutinoside	3.97	594.16	$[M+H]^+$	595	287	−31	449	−20
3,4-Dihydroxybenzoic acid	2.09	154.03	$[M-H]^-$	153	109	16	108	25
Ellagic acid	5.51	302.01	$[M-H]^-$	301	145	38	284	29
Epigallocatechin gallate	4.77	458.08	$[M-H]^-$	457	169	21	125	38
trans-Ferulic acid	5.67	194.06	$[M-H]^-$	193	134	15	178	15
Gallic acid	1.02	170.02	$[M-H]^-$	169	125	16	79	23
Myricetin	6.74	318.04	$[M-H]^-$	317	151	25	179	18
Quercetin	7.78	302.04	$[M+H]^+$	303	153	−32	137	−31
trans-Resveratrol	7.06	228.08	$[M+H]^+$	229	107	−25	135	−15
Rutin	5.68	610.15	$[M-H]^-$	609	300	40	301	33

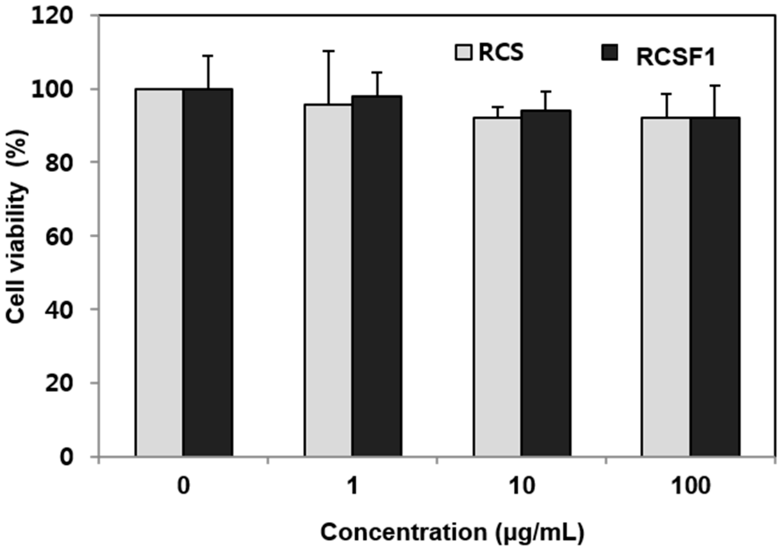

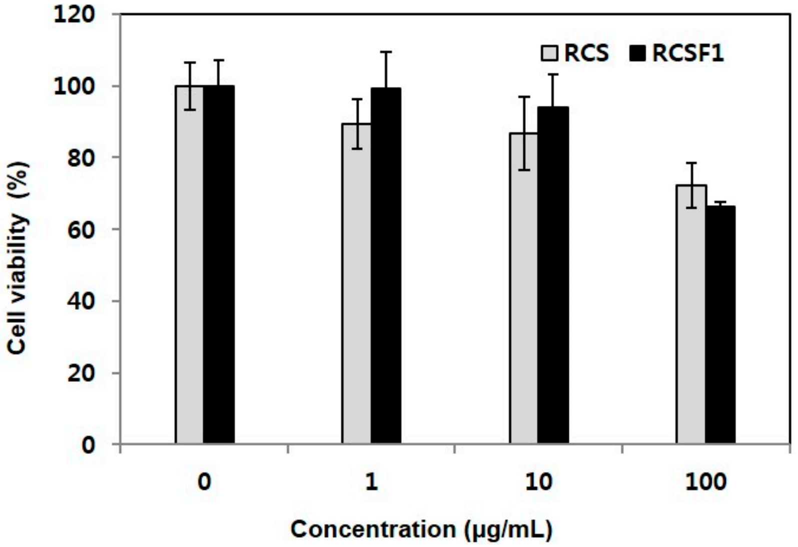

Figure S1. Cytotoxicity of RCS and RCSF1. Cytotoxicities of RCS and RCSF1 were measured by MTT assay. MDCK cells were treated with RCS or RCSF1 for 24 h (**upper panel**) and 48 h (**lower panel**). The percentage of cell viability was calculated as follows: % cell viability = ($Abs_{treatment}$ / $Abs_{control}$) × 100.

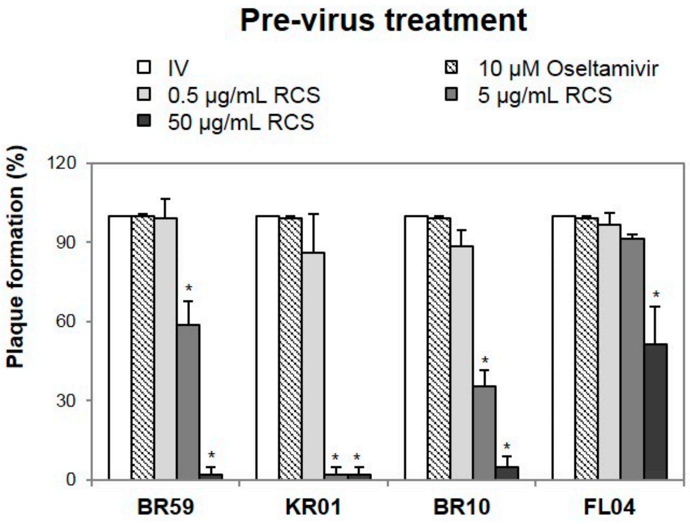

Figure S2. *Cont.*

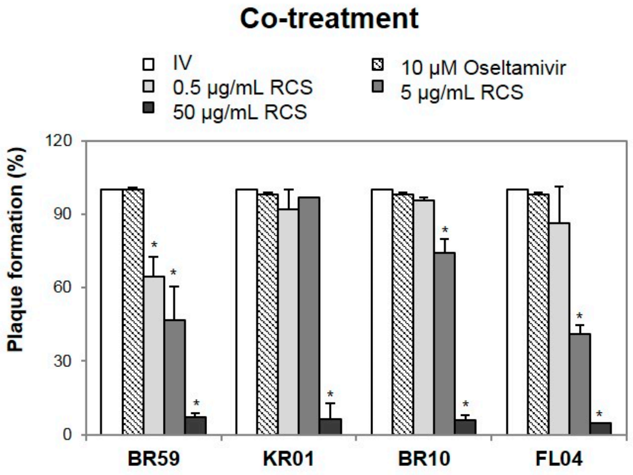

Figure S2. Antiviral activities of RCS against BR59, KR01, BR10, and FL04. In time-of-addition assays, pre-virus treatment (**upper panel**): each virus was mixed with RCSF1 for 1 h prior to viral infection. Co-treatment (**lower panel**): cells were infected with virus and simultaneously treated with RCS for 1 h at 37 °C. After infection, the cell monolayers were washed with PBS and overlaid with DMEM and agarose at 37 °C for 72 h. Plaques were counted after 0.5% crystal violet staining. Non-treated influenza virus (IV) and oseltamivir (10 μM) were used as negative and positive controls, respectively. All measurements were performed in triplicate. An asterisk (*) denotes a significant decrease in plaque formation relative to the control ($p < 0.05$).

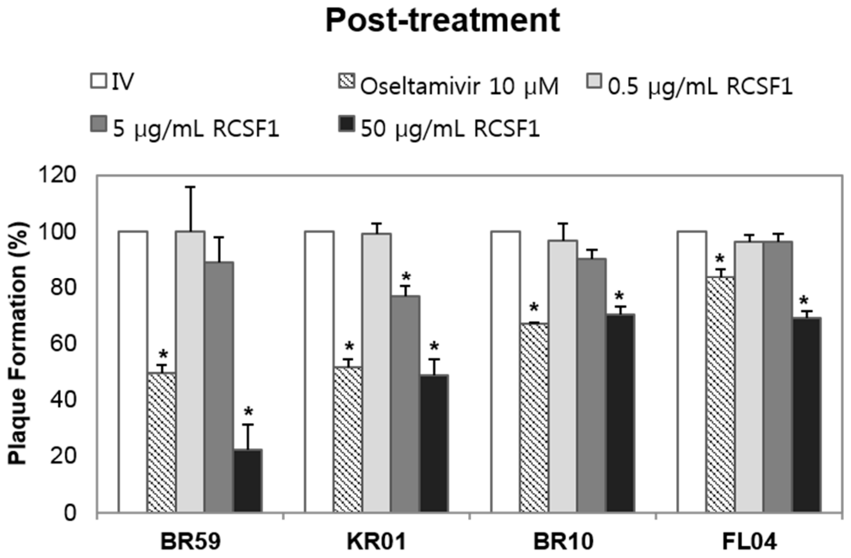

Figure S3. Antiviral activities of RCSF1 against BR59, KR01, BR10, and FL04 in the post-treatment. For post-treatment, virus was absorbed to cells at 37 °C for 1 h, which were treated with RCSF1 for 1 h and overlaid with DMEM containing trypsin and agarose for 72 h. Plaques were counted after crystal violet staining. Non-treated influenza virus (IV) and oseltamivir (10 μM) were used as negative and positive controls, respectively. All measurements were performed in triplicate. An asterisk (*) denotes a significant decrease in plaque formation relative to the control ($p < 0.05$).

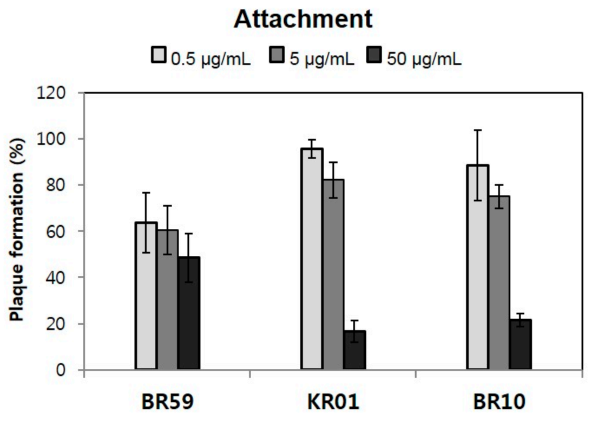

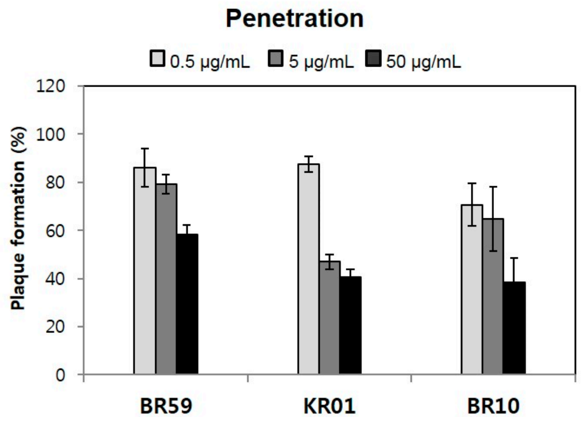

Figure S4. Inhibitory effects of RCSF1 on attachment or penetration into host cells. For attachment assay (**upper panel**), chilled MDCK cells were infected with virus and simultaneously treated with RCSF1 (0.5–50 µg/mL) for 1 h at 4 °C. After infection, the supernatant was removed and cells were washed twice with ice-cold PBS. For penetration assay (**lower panel**), chilled MDCK cells were infected with virus at 4 °C for 1 h. MDCK cells were treated with RCSF1 (0.5–50 µg/mL) at 37 °C for 1 h. After the supernatant was removed, un-penetrated virus was inactivated by acidic PBS (pH 3) and neutralized with alkaline PBS (pH 11) and washed with ice-cold PBS. Cells were overlaid with DMEM containing TPCK-trypsin and agarose for 72 h at 37 °C, 5% CO_2. Plaques were counted after 0.5% crystal violet staining. The plaque formation of influenza virus-infected MDCK cells was 100%. All measurements were performed in triplicate.

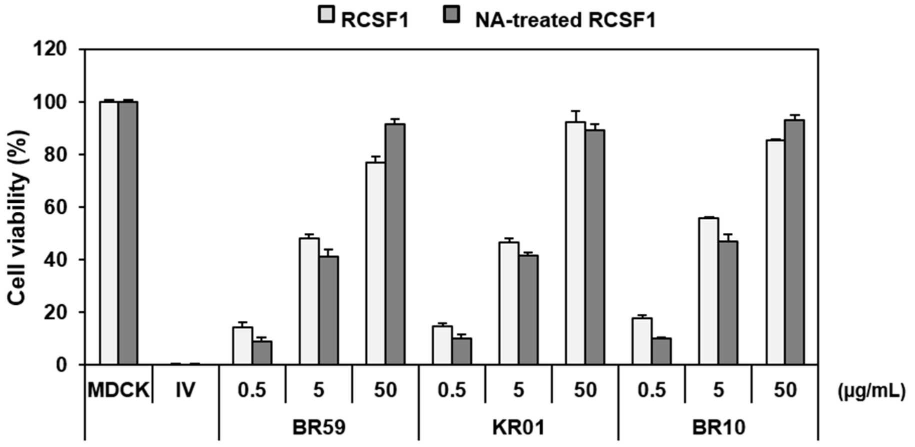

Figure S5. Effect of NA treatment on RCSF1. MDCK cells were infected with virus at an MOI of 0.001 for 1 h at 37 °C. After removing the medium, the cells were treated with RCSF1 or NA-treated RCSF1 (1 unit NA per 1 µg RCSF1) which was serially diluted in DMEM containing trypsin. After incubation for 72 h at 37 °C and 5% CO_2, the cell viability was measured using MTT assay.

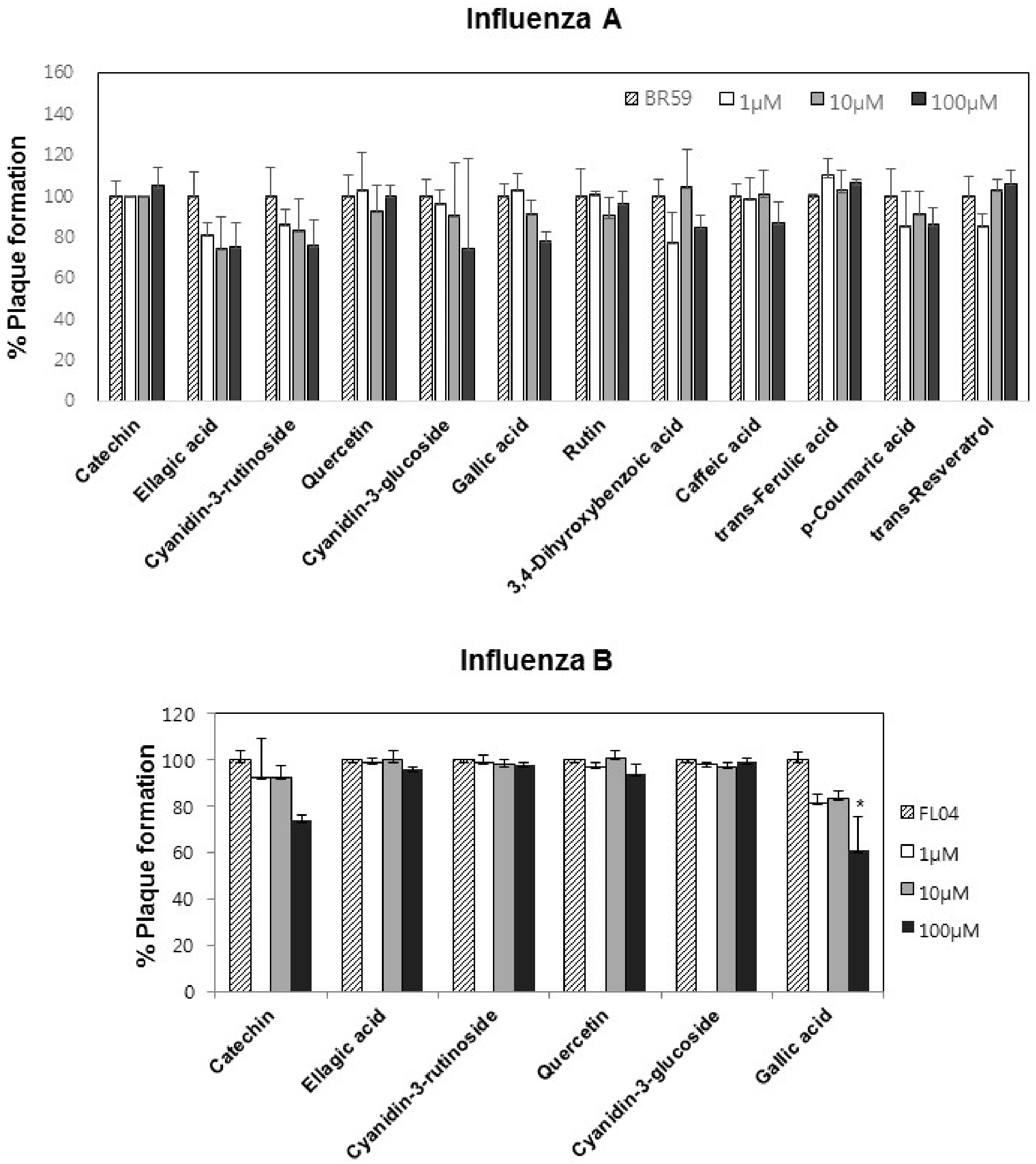

Figure S6. Antiviral activities polyphenols against influenza type A and B in the co-treatment. Cells were infected with virus and simultaneously treated with polyphenol for 1 h at 37 °C. After infection, the cells were washed with PBS and overlaid with DMEM and agarose at 37 °C for 2–3 d. Plaques were counted after crystal violet staining. Non-treated influenza virus (IV) and oseltamivir (10 μM) were used as negative and positive controls, respectively. Co-treatment with oseltamivir exhibited no inhibition against influenza type A and B. All measurements were performed in triplicate. An asterisk (*) denotes a significant decrease in plaque formation relative to the non-treated influenza virus ($p < 0.05$).

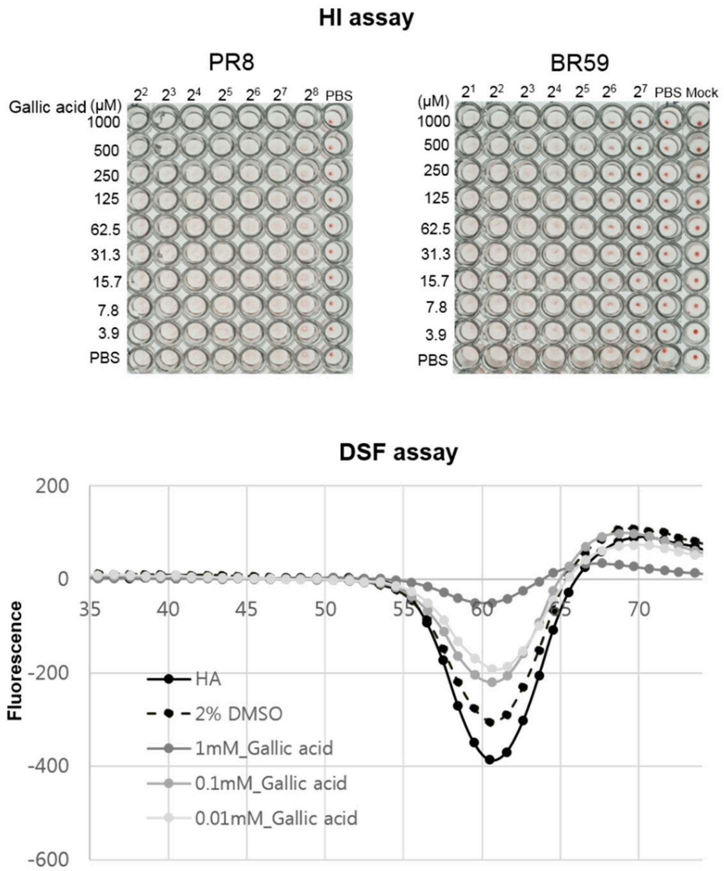

Figure S7. Effects of gallic acid on hemagglutination and binding to hemagglutinin. For HI assay (**upper panel**), gallic acid (3.9–1000 μM) was incubated with 2-fold serial diluted virus and chicken red blood cells for 1 h before hemagglutination inhibition was determined. For DSF assay (**lower panel**), the melting temperature T_m profile of influenza virus HA in the absence and presence of gallic acid (0.01–1 mM) was measured.

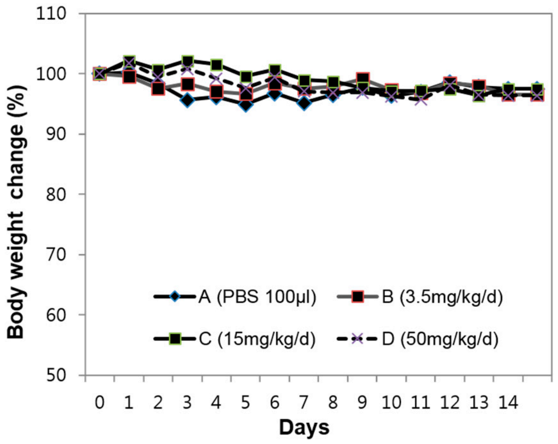

Figure S8. *In vivo* toxicity of RCSF1. Six-week-old BALB/c mice (five per group) were orally treated with RCSF1 at doses of 0, 3.5, 15, and 50 mg/kg/day for 8 days. Body weight changes of mice were measured daily for 14 days.

© 2016 by the authors; licensee MDPI, Basel, Switzerland. This article is an open access article distributed under the terms and conditions of the Creative Commons by Attribution (CC-BY) license (http://creativecommons.org/licenses/by/4.0/).
